# White Matter Diffusivity Predicts Change in Sight Word Reading Following Continuous Theta Burst Stimulation to the Left Temporal Parietal Junction

**DOI:** 10.1162/NOL.a.217

**Published:** 2026-02-20

**Authors:** C. Nikki Arrington, Ewelina Bledniak, Brianna Kinnie, Evelyn Farkas, Robin Morris, Fumiko Hoeft

**Affiliations:** Department of Psychology, Georgia State University, Atlanta, GA, USA; Department of Psychological Sciences, University of Connecticut, Storrs, CT, USA; Medical College of Georgia, Augusta University, Augusta, GA, USA; Department of Psychology, Florida State University, Tallahassee, FL, USA

**Keywords:** neuromodulation, reading efficiency, transcranial magnetic stimulation (TMS), word reading

## Abstract

Successful coordination of the functional networks underlying reading is highly dependent on the underlying white matter tracts that connect these regions. Continuous theta burst stimulation (cTBS) can temporarily inhibit brain activity in targeted brain networks and has been shown to modulate reading ability. It was hypothesized that measures of diffusivity would predict change in reading efficiency following stimulation to the left temporal-parietal junction (TPJ), a targeted node of the dorsal stream of the reading network. Fifty-three adults between the ages of 18 and 50 years (*M* = 22.79, *SD* = 5.40; 34 female) with a range in reading ability completed sight word and pseudoword fluency measures prior to and immediately following cTBS to either the left or right TPJ or a control site. Participants also completed an MRI session including anatomical and diffusion weighted imaging sequences. Regression analyses were conducted to predict change in reading fluency following cTBS. Tracts associated with the reading network including the arcuate fasciculus, inferior longitudinal fasciculus, and portions of the superior longitudinal fasciculus, as well as the corpus callosum significantly predicted reliable change in a test of word reading efficiency–sight word efficiency subtest following stimulation of the left TPJ. These findings suggest that increased diffusivity of white matter tracts associated with the left hemisphere reading network and their right hemisphere homologues may support the impact of cTBS following stimulation to a targeted node of the reading network. Individual differences in white matter diffusivity may underlie differences in behavioral outcomes following neuromodulation.

## INTRODUCTION

Reading involves a sophisticated orchestration of phonological, semantic, and visual processes. An individual must be able to effectively integrate all three modalities for the identification of novel words. Beyond word recognition, text comprehension requires cognitive processes that support causal inference and fluent reading. Learning to read depends on the interplay of multiple cognitive and linguistic skills, such as phonological awareness, accuracy, and fluency (Rayner et al., 2001; Schlaggar & McCandliss, 2007), and unfolds over time as part of a multifaceted developmental process. Prior to the onset of fluent reading, these foundational skills are shaped by both print exposure and formal instruction, while continuing to evolve alongside brain maturation (Houston et al., 2014). This developmental progression places significant demands on a range of neurocognitive systems, requiring the recruitment and integration of multiple developing neural systems (Pugh et al., 2000; Rueckl et al., 2015).

The two major aspects of reading acquisition—speed and accuracy—directly affect reading fluency and efficiency. With repeated practice, word and letter patterns become consolidated in long-term memory, enabling them to be processed as a single unit. This process builds a reader’s schema for automatic decoding, a critical contributor to rapid and accurate word recognition (LaBerge & Samuels, 1974; Logan, 1997; Nagy et al., 1987). Efficiency in phonological processing (speed and accuracy), particularly of nonwords, serves as a robust indicator of reading proficiency independent of vocabulary knowledge (Silverman et al., 2013). Beyond phonological decoding and automatic word recognition, verbal expression emerges as an important component of oral reading fluency, one that significantly improves through practice and continues to develop throughout the early elementary years (Schwanenflugel et al., 2004).

### Neural Substrates of Reading

Reading is a uniquely human capability that involves the successful coordination of multiple brain regions. Research has identified a distinct, left-lateralized network that supports reading (Murphy et al., 2019). Functional neuroimaging studies have highlighted three major neural pathways that make up the reading network: anterior, dorsal, and ventral systems (Pugh et al., 2000). The ventral pathway, which supports orthographic-pattern processing and word-reading automaticity, includes the left occipitotemporal cortex and the inferior and the middle temporal gyri (Schlaggar & McCandliss, 2007; Shaywitz et al., 2004). The dorsal pathway comprises regions in and around the angular gyrus, including the inferior parietal lobule and supramarginal gyrus, as well as posterior portions of the superior temporal gyrus associated with Wernicke’s area. This pathway aids in phonological processing and decoding of single words (Cohen et al., 2000, 2002). Finally, the anterior pathway, centralized around Broca’s area and including the inferior frontal gyrus (IFG), is thought to be involved in complex aspects of reading such as text processing and reading comprehension (Shankweiler et al., 2008; Vigneau et al., 2006).

While fluent reading of regular words relies heavily on orthographic information processed by the ventral pathway, pseudoword reading, or the reading of unknown novel words, typically requires active phonological decoding via the dorsal pathway (Shaul et al., 2012). Developed readers show this distinct pattern of neural activation when reading real words compared with pseudowords (Hagoort et al., 1999; Shaul et al., 2012). This same differentiation is not seen in those with developmental reading impairments.

#### White matter tracts supporting word reading

Successful coordination of the functional networks underlying reading is highly dependent on the underlying white matter tracts that connect these regions (Vandermosten, Boets, Wouters, & Ghesquière, 2012). In typical development, the maturation of these tracts has been associated with the acquisition of reading skills (Borchers et al., 2019; Myers et al., 2014; Travis et al., 2017; Vanderauwera et al., 2018). Mean fractional anisotropy (FA), which quantifies directionality of water diffusion along the tract, has been shown to correlate with behavioral measurements of reading, spelling, and rapid naming, providing support for the left hemisphere neural network necessary for fluent reading (Arrington et al., 2017; Deutsch et al., 2005; Vandermosten, Boets, Poelmans, et al., 2012; Yeatman et al., 2011). Lower mean diffusivity (MD), an average measurement of water diffusion, in regions associated with the left-lateralized reading network have been associated with better phonological decoding and reading comprehension skills (Martins et al., 2024; Partanen et al., 2021). Decreases in MD in white matter tracts within the reading network have also been associated with improved reading ability following an intensive intervention, suggesting a direct link between white matter plasticity and reading development (Meisler et al., 2024). Higher FA in tracts associated with the dorsal pathway, including the left arcuate fasciculus (AF) and superior longitudinal fasciculus (SLF), has been correlated with phonological aspects of reading, such as pseudoword reading, in both children and adults (Gullick & Booth, 2015; Horowitz-Kraus et al., 2014; Qiu et al., 2011; Travis et al., 2017; Yeatman et al., 2011). Reduced FA values within the SLF have been reported in adults with poor word reading ability such as those seen in developmental dyslexia, which in turn supports the role of the SLF in phonological processing (Carter et al., 2009; Vandermosten, Boets, Poelmans, et al., 2012). White matter tracts associated with the ventral and anterior pathways, namely, the inferior longitudinal fasciculus (ILF) and uncinate fasciculus (UF), have also been associated with orthographic components of reading (i.e., word recognition; Arrington et al., 2017; Cummine et al., 2015; Wang et al., 2020).

Additionally, micro- and macrostructural properties of the corpus callosum (CC) have been shown to relate to reading ability (Fine et al., 2007; Hasan et al., 2012). Reading and writing training has also been shown to change morphological properties of parietal regions of the CC in adults without previous formal literacy instruction (Castro-Caldas et al., 1999). Differences in micro- and macrostructural properties of posterior regions of the CC have also been reported in children and adults with developmental reading impairments (Hasan et al., 2012; Rumsey et al., 1996; von Plessen et al., 2002), with reduced MD observed in children with specific fluency deficits (Hasan et al., 2012).

### Neuromodulation of the Reading Network

Transcranial magnetic stimulation (TMS) is a noninvasive brain stimulation technique that can produce short-term changes in cortical excitability in targeted brain regions (Hallett, 2007; Rossini et al., 2015). During TMS, a transient magnetic pulse induces a magnetic field which passes into the cortex and induces electrical changes in the targeted cortical region (Walsh & Rushworth, 1999). TMS can have a faciliatory or suppressive effect depending on the rate of stimulation. Magnetic stimulation induces electrical changes in the cortical regions in relation to white matter fiber that connect to downstream brain regions. It can in turn lead to the alteration of membrane potentials along the fiber tracts (Ferreri et al., 2011; Hannula & Ilmoniemi, 2017). This alteration then facilitates neural activity propagation in the brain when TMS is applied.

Repetitive TMS consists of the application of brief trains of patterned pulses to a targeted brain region. Effects of repetitive TMS varies depending on the stimulation protocol used (i.e., frequency and duration). Various repetitive TMS paradigms have been shown to result in effects on cortical activity that last beyond the stimulation period. Repeated sessions of repetitive TMS can lead to lasting changes in cognitive and affective processing (Hallett, 2020; Rossini et al., 2015). Continuous theta burst stimulation (cTBS) is a specified repetitive stimulation paradigm that consists of patterned bursts of three pulses at 50 Hz given at 5 Hz, for 40 s (Hallett, 2020; Huang et al., 2005). Like traditional repetitive paradigms, TBS produces prolonged effects on cortical excitability, which can lead to changes in behavior that are believed to be driven by changes in long-term depression. cTBS has been shown to produce changes in behavior lasting up to 1 hr post stimulation, following much shorter durations of stimulation than traditional repetitive TMS paradigms (Harrington et al., 2023; Lefaucheur, 2019).

Neuromodulation techniques, such as TMS, have the potential to create short-term, yet systematic changes in brain regions associated with the reading network (Arrington et al., 2022). As such, TMS provides the opportunity to better understand the neural basis of typical and impaired reading. Stimulation to targeted nodes within the dorsal pathway of the reading network has been shown to distinctly modulate phonological processing (Gough et al., 2005; Harrington et al., 2023; Sliwinska et al., 2012; Stoeckel et al., 2009). A single session of cTBS to the supramarginal gyrus has been shown to facilitate phonological processing for up 60 min post stimulation (Harrington et al., 2023). High frequency repetitive TMS (5 Hz) to the left inferior parietal lobule has been shown to decrease non-word reading errors in typically developed readers (Costanzo et al., 2012). A similar stimulation paradigm delivered to the left or right inferior parietal lobule improved pseudoword reading in adults with developmental dyslexia (Costanzo et al., 2013). Conversely, repetitive 10 Hz TMS to a node of the dorsal pathway was shown to significantly slow reading speed for a list of real words, compared to stimulation to the anterior pathway (Leff et al., 2001).

Semantic processing and single word reading are also selectively impacted following stimulation to nodes within the ventral and anterior pathways or their right hemisphere homolog (Carreiras et al., 2012; Chouinard et al., 2009; Costanzo et al., 2012, 2013; Pattamadilok et al., 2015; Wheat et al., 2013; Whitney et al., 2011). High frequency repetitive TMS (5 Hz) to the left superior temporal gyrus improved single word reading speed in a group of impaired readers (Costanzo et al., 2013). Interestingly, high frequency TMS, greater than 5 Hz, to the ventral occipital temporal cortex decreased reading speed and increased errors when responding to real words in typical readers (Lavidor et al., 2003; Lavidor & Walsh, 2003). Alternatively, cTBS to the middle temporal gyrus had no effect on sentence reading (Acheson & Hagoort, 2013). Taken together, these results would suggest that the impact of TMS on reading is highly dependent on stimulation site and frequency.

Although the electrical field is primarily induced in cortical regions directly under the TMS coil, effects of stimulation can be observed in regions connected downstream of the targeted node (Esposito et al., 2020; Ferreri & Rossini, 2013; Hannula et al., 2010; Siebner et al., 2022). As such, e-field modeling, in combination with structural connectivity measures, provides a reliable model of TMS effects on neural networks (Geeter et al., 2016). Research has indicated that white matter structure is an important factor in determining behavioral response following neuromodulation. Specifically, regional FA values account for individual differences in TMS-induced measures of cortical excitability (Klöppel et al., 2008). Additionally, regional FA and MD values have been linked to functional TMS outcomes (Kearney-Ramos et al., 2018; Neubert et al., 2010). This highlights the importance of better understanding the relation between TMS outcomes and white matter underlying targeted networks for reading.

### Current Study

Although neuromodulation techniques are increasingly used to investigate the neural mechanisms of reading, no study to date has examined the link between properties of white matter microstructure and reading outcomes following TMS. The current study aimed to assess the relation between measures of diffusivity (i.e., FA and MD) for tracts associated with the reading network and TMS-induced changes in real word versus pseudoword reading efficiency. Modulation of the left temporal-parietal junction (TPJ), a key node within the dorsal system, was expected to impact reading efficiency through white matter pathways connecting the reading system. We hypothesized that measures of diffusivity within left-lateralized reading tracts would be correlated with changes in reading efficiency following stimulation of the left TPJ. Specifically, we expected to see a stronger relation between change in pseudoword reading, compared to reading of real words, and white matter diffusivity in dorsal pathways due to the downregulation of the dorsal system. Conversely, we expected no such relations following stimulation of the right TPJ or the vertex.

## MATERIALS AND METHODS

### Participants

This investigation utilized a subset of data collected from a larger study investigating neural mechanisms underlying compensation in dyslexia. Specifically, the analysis focused on participants who: (a) successfully obtained diffusion weighted-imaging (DWI), (b) were administered the Tests of Word Reading Efficiency (TOWRE-2) Forms A and B (Torgesen et al., 2012) on the same day before and after the TMS session (described below), and (c) received TMS to either the vertex or the left/right temporoparietal region.

Fifty-three participants between the ages of 18 and 50 years (*M* = 22.79, *SD* = 5.40; 34 female) were selected from the larger dataset based on the above criteria. All were native or native-like speakers of English, participated in the larger study after providing informed consent, and received monetary compensation in exchange for participation. Participants were recruited from the Storrs, CT, and Atlanta, GA, regions via posts on social media and partnerships with local organizations willing to advertise on behalf of the research project, as well as flyers distributed to surrounding learning disability organizations, junior colleges, community colleges, state universities, libraries, and literacy centers. The protocol was approved by the University of Connecticut Institutional Review Board.

All participants had no reported history of major neuropsychiatric illness (e.g., severe mood disorder, alcoholism), were not actively receiving intensive reading remediation, and had no contraindications to magnetic resonance imaging (MRI) or TMS such as metallic hardware in or on their body (e.g., cochlear implants, medication pumps, pacemakers, brain stimulators). Further exclusion criteria included a history of seizure, epilepsy, stroke, brain surgery, severe head injury, cranial metal implants, known structural brain lesions, and devices that may be affected by TMS. Criteria for TMS were largely based on current safety guidelines (Rossi et al., 2021).

To ensure eligibility, potential participants were screened for intellectual deficits using the Wechsler Abbreviated Scale of Intelligence II (WASI-II) (Wechsler, 2011). Only participants with a WASI-II Full-Scale Intelligence Quotient (FSIQ) score—composed of the Vocabulary and Matrix Reasoning subtests—greater than 80 were eligible for the study. Participants were also screened for timed word reading ability using the TOWRE-2. Participants completed a survey that contained questions regarding self-reported reading and language development including the Adult Reading History Questionnaire (ARHQ; Lefly & Pennington, 2000). Those who were enrolled in the study went on to complete a battery of neuropsychological assessments, described below.

### Reading Measures

To assess baseline reading ability, the Woodcock-Johnson IV Tests of Achievement (WJIV-ACH) Letter-Word Identification (LWID) test of single word reading and the Word Attack (WA) test of single pseudoword reading were administered to obtain the WJIV-ACH Basic Reading Cluster Score (Schrank et al., 2014). The included tests of the WJIV-ACH have been shown to be a valid measure of reading ability in this age range (Villarreal, 2015).

### Experimental Task

Prior to receiving stimulation, participants completed the TOWRE-2 Form A as a measure of baseline reading efficiency, in which participants were instructed to read a list of either real words or pseudowords both quickly and accurately (Torgesen et al., 2012). The Sight Word Efficiency (SWE) subtest served as a measure of sight word reading efficiency while the Phonemic Decoding Efficiency (PDE) subtest served as a measure of pseudoword reading efficiency. All participants completed both TOWRE-2 SWE and PDE subtests. Each subtest for TOWRE-2 Form B was administered after TMS immediately following stimulation. Alternate forms of each subtest of the TOWRE-2 maintains an average reliability coefficient for the subtests exceeding 0.90. The average test–retest coefficients for different forms of the subtests are 0.87, making them ideal for repeated measures assessment. Reliable change scores were calculated for SWE and PDE following stimulation of the left and right TPJ, as well as a control site (vertex; Zahra, 2010). The reliable change index (RCI) accounts for reliability in validated measures such as TOWRE-2 and standardizes change scores using related standardized units.

### Transcranial Magnetic Stimulation

Stimulation was applied using a figure-of-eight magnetic coil (MCF-B65) connected to MagVenture MagPro X100 Magnetic Stimulator. Inhibitory cTBS consisted of a series of three-pulse bursts given at 50 Hz repeated for a total of 600 pulses lasting for 40 s (Huang et al., 2005). cTBS was delivered at 80% of the individual-determined active motor threshold as determined using maximum-likelihood parameter estimation by sequential testing (Ah Sen et al., 2017) based on standard procedures (Rossini et al., 2015).

Participants received stimulation to at least one of the following brain regions: left TPJ, right TPJ, or vertex. Only one brain region was stimulated during each TMS session. Some participants completed two TMS sessions on different days and thus received stimulation to more than one brain region. Order of stimulation site was randomized. Targets were localized using contrast maps from the functional phonological-orthographic decision task completed during their baseline visit (see below). Vertex was identified using anatomical landmarks (midline at the central sulcus). Stimulation target sites were localized using a stereotactic MRI-guided Localite neuronavigation system.

### Experimental Procedure

Participants completed the study over the course of several sessions with the days containing TMS stimulation being at least 2 weeks apart (*M* = 57.11 days, *SD* = 58.74). During a baseline session, participants completed the battery of neuropsychological assessments as well as a 1-hr MRI scan during which they (1) watched a movie while we obtained a T1w anatomical image to be used for neuronavigation and (2) completed two runs of a timed phonological-orthographic decision task.

Following the baseline session (*M* = 151.29 days, *SD* = 156.12), each participant underwent at least one additional session that included TMS stimulation of the left or right TPJ or vertex (Session 1: 10 LTP, 16 RTP, 9 vertex; Session 2: 10 LTP, 9 RTP, 15 vertex). Sixteen participants completed an additional session on a different day. Table 1 provides the distribution of stimulation sites for those participants who completed two sessions.

**Table 1.** Distribution of stimulation site and order of stimulation for 16 participants who completed more than one study session

		Second TMS session		
		LTP	RTP	Vertex
First TMS session	LTP	0	1	5
	RTP	4	0	2
	Vertex	2	2	0

*Note*: LTP = left temporoparietal region; RTP = right temporoparietal region.

Prior to the start of TMS stimulation, participants were administered TOWRE-2 Form A SWE and PDE. Immediately following cTBS, participants completed both subtests of TOWRE-2 Form B. During one session, after completing the post-stimulation reading tasks, participants completed an MRI scan where they watched a movie of their choice while diffusion-weighted imaging was obtained.

### MRI Scan Sequences

#### Anatomical imaging

Prior to collecting diffusion-weighted images, 0.8 × 0.8 × 0.8 mm high-resolution, three-dimensional T1-weighted sagittal acquisition was acquired (MPRAGE pulse sequence; TE: 2.22 ms; TR: 2,440 ms; FA: 8°; FOV: 256 × 256 pixels; slice thickness 0.8 mm; 208 slices). T2*-weighted images were collected in the same orientation (matrix size = 32 × 32; voxel size = 0.8 × 0.8 × 0.8 mm; FOV = 256 mm; TR = 3,200 ms; TE = 563 ms).

#### Task fMRI

Functional volumes were acquired using a T2*-weighted gradient-echo EPI sequence with the following parameters: TR = 1,630 ms, multiband accelerated factor = 3 (TE1 = 16.00 ms, TE2 = 37.79 ms, TE3 = 59.58 ms) flip angle = 67°, FOV = 210 mm, voxel size = 2.5 × 2.5 × 2.5 mm^3^, with 54 slices acquired in transversal orientation and phase encoding direction A/P. B0 field maps were acquired for susceptibility distortion correction (TR = 6,000 ms, deltaTE = 52.80 ms, flip angle = 90° with other parameters identical to the functional sequence).

Participants completed two runs of the functional task. A mixed design was used, consisting of three rhyming task blocks (each 56 s), two matching task blocks (each 40 s), and rest blocks (each 14 s) placed between task blocks. Before each task block, a prompt appeared at the center of the screen for 2 s, indicating the task type (“Do the words rhyme?” or “Are the letter strings the same?”). On each trial, two high-frequency real words were visually presented simultaneously above and below fixation for 2 s, followed by a jittered blank screen lasting 1 to 2 s. Participants responded as quickly and accurately as possible using their right hand: the index finger was used for “rhyming/matching” responses, and the middle finger for “non-rhyming/non-matching” responses. Each rhyming block contained 16 trials, and each matching block contained 12 trials. Half of the trials required a “yes” response, and the trial order was pseudorandomized.

**Figure 1.**
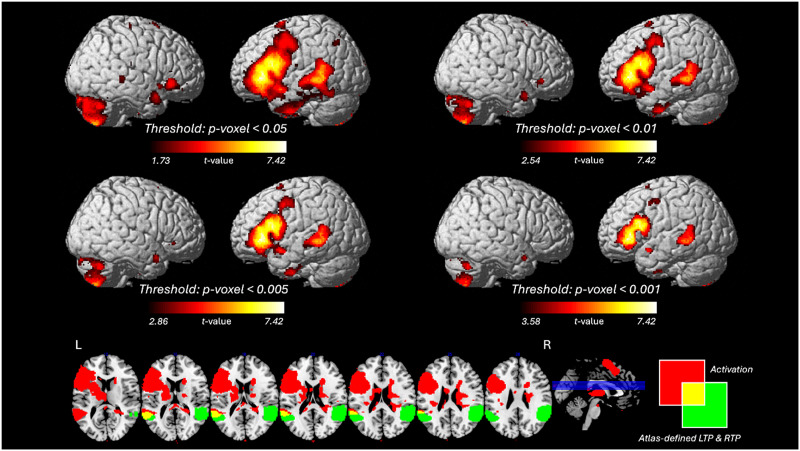
Peak loci for functional phonological-orthographic decision task used for transcranial magetic stimulation (TMS) targeting. LTP = left temporoparietal region; RTP = right temporoparietal region.

Preprocessing and analysis of the fMRI task data were conducted using AFNI (Version 12.0.03; RRID:SCR_005927; Cox, 1996; Cox & Hyde, 1997) and FSL (Version 6.0.5; RRID:SCR_002823; Jenkinson et al., 2012; Smith et al., 2004). Before preprocessing, the three echoes were combined with Multi-Echo Independent Components Analysis (ME-ICA), which was included in AFNI (https://afni.nimh.nih.gov/pub/dist/src/pkundu/README.meica). The following preprocessing steps were conducted with the output images in the native space: motion correction with MCFLIRT, BET, spatial smoothing with a 5-mm full-width half maximum kernel, and high-pass temporal filtering with a cutoff of 90 s. Six standard rotation and translation motion parameters were included in the hemodynamic response function modeling, and brain activation maps were generated for the contrast “rhyming > matching.” *Z* scores were produced from permutation tests with 5,000 iterations. The peak activation loci within the left and right temporoparietal regions—defined as the union of the supramarginal and angular gyri in the Harvard–Oxford atlas (RRID:SCR_001476) and wrapped into the native space—were identified (see Figure 1). These peak loci were used as targets for TMS stimulation. If no voxel was detected with a threshold of *z* score > 0.5 within the anatomical mask or the peak loci were too far off from the central area of the regions of interest, the MNI-based center of mass of the anatomical region was used as an alternative (left TPJ = 4, right TPJ = 9). The decision was made and reviewed by at least three team members who were blinded to the analysis.

#### Diffusion-weighted imaging

Diffusion-weighted images were acquired to characterize properties of white matter diffusivity (FOV = 192 × 192 mm^2^, matrix = 110 × 110, 66 transverse slices, 2 mm isotropic resolution, anterior-to-posterior (AP) phase encoding, TR = 3,894 ms, TE = 88.00 ms, FA = 90°, acquisition BW = 1,488 Hz/pixel, 9 b = 0 s/mm^2^ images, 210 directions at b = 2,500 s/mm^2^). A posterior-to-anterior (PA) phase encoding b = 0 s/mm^2^ volume was collected to correct for EPI-induced distortions during processing.

Images were acquired using a 3T Siemens scanner located at the GSU/GaTech Center for Advanced Brain Imaging (Atlanta) or the Brain Imaging Research Center (Storrs). Data acquisition and scan parameters were kept consistent across sites. To account for differences in data collected between sites, tract level FA and MD average weighted values were harmonized using ComBat (Johnson et al., 2007). ComBat assumes the imaging feature measurements can be modeled as a linear combination of the biological variables (i.e., age, sex, and baseline reading ability) with the scanner effects as an error term that includes a multiplicative scanner-specific scaling factor. It has been shown to effectively reduce interscanner variation in neuroanatomical data while effectively preserving biological associations (Fortin et al., 2017).

#### Imaging quality assessment and data preprocessing

All data received visual and automated quality assurance to assess for signal dropout and motion artifact. Data passing initial quality assurance was then processed through in-house automated pipelines as outlined below. Anatomical data was processed using FreeSurfer 7.2.0 image analysis suite (Dale et al., 1999; Fischl, 2012) which is documented and freely available for download online (https://surfer.nmr.mgh.harvard.edu/). This automated procedure contained segmentation of cortical and subcortical white matter, tessellation of gray matter/white matter boundaries, inflation of the folded surface tessellation patterns (Fischl, Sereno, & Dale, 1999; Fischl, Sereno, et al., 1999), and automatic correction of topographical defects (Fischl & Dale, 2000). Visual quality assurance was completed by a trained technician based on best practices (Monereo-Sánchez et al., 2021).

DWI data underwent automated quality assurance utilizing DTIprep, which quantifies motion artifact, b0 alignment, and useable gradients (Oguz et al., 2014). Participants whose scans failed this quality control checkpoint were not included in these analyses (*n* = 5). Data was preprocessed using MRtrix 3.0 (Tournier et al., 2019), which is an open-source software (https://www.mrtrix.org/download/) that allows for processing, visualization, and analysis of diffusion MRI data. MRtrix allows for refinement of multishell DWI data for probabilistic tractography. Raw diffusion data were denoised using the dwidenoise command which implements a noise level estimation and denoising based on random matrix theory (Cordero-Grande et al., 2019). Denoised data were corrected for motion and eddy current-induced distortion using eddy_cudda (Andersson et al., 2017) as part of the FSL FMRIB’s Diffusion Toolbox. Eddy corrected data were assessed for quality using eddy QC tools (Andersson et al., 2016; Bastiani et al., 2019) with the percentage of total outliers recorded. Finally, B1 field inhomogeneity correction was performed on distortion corrected data using the MRtrix dwibiascorrect fsl command.

Automated reconstruction of white matter tracts of interest was completed using the FreeSurfer’s TRACULA (TRActs Constrained by UnderLying Anatomy), global probabilistic tractography pipeline (Maffei et al., 2021; Yendiki et al., 2011). TRACULA’s default tensor fitting and tract reconstruction were applied to the preprocessed data. Tract reconstruction was conducted using the BEDPOSTX ball-and-stick model applied to the preprocessed data to estimate diffusion probability. The combination of FreeSurfer’s cortical parcellation and subcortical segmentation with TRACULA’s anatomical atlas provided the automated reconstruction of white matter tracts. Average stream-line weighted FA and MD values were extracted for white matter tracts associated with the left hemisphere reading network and their right hemisphere homologs: AF, ILF, SLF (segments I, II, and III), and UF as well as regions of the CC (parietal, prefrontal, premotor, temporal bodies, genu, rostrum).

### Statistical Analyses

Participants with individual sessions where SWE or PDE change scores were outside 2.5 standard deviations were considered to be outliers and were removed from the sample. As such, two participants were excluded from the sample (1 RTP, 1 vertex). Final analyses included 51 participants with 67 usable sessions (Table 2).

**Table 2.** Mean standard scores pre-/post-TMS for TOWRE-2 by stimulation site

Site	*N*	Pre-TMS (TOWRE-2 Form A)		Post-TMS (TOWRE-2 Form B)	
		SWE SS Mean (*SD*)	PDE SS Mean (*SD*)	SWE SS Mean (*SD*)	PDE SS Mean (*SD*)
LTP	20	108.45 (15.18)	101.45 (10.78)	103.45 (25.66)	101.4 (24.24)
RTP	24	108.04 (27.02)	103.33 (25.02)	104.04 (12.08)	102.71 (14.44)
Vertex	23	107.65 (12.95)	107.04 (12.27)	104.30 (13.43)	106.39 (9.79)

*Note*: TMS = transcranial magnetic stimulation; LTP = left temporoparietal region; RTP = right temporoparietal region; SWE = TOWRE-2 Sight-Word Efficiency; PDE = TOWRE-2 Phonemic Decoding Efficiency; *SD* = standard deviation; SS = standard score.

Analyses were performed using IBM SPSS Statistics (Version 29.0.1.1). SWE and PDE RCI was derived from post- minus pre-TMS subtest standard scores, with SWE and PDE calculated separately to reflect change in single word and pseudoword reading efficiency following TMS stimulation. Negative RCI values were indicative of poorer performance post stimulation as indicated by a lower standard score on a given subtest. Independent one-way analyses of variance were performed to investigate differences in reliable change based on stimulation site. Tukey’s HSD Test was used to identify pairwise differences.

In addition, Pearson’s correlation analyses were conducted to assess relations between DWI metrics for white matter tracts implicated in reading and reliable change in timed word and non-word reading measures. Separate correlations were run for each of the three stimulation sites. Relations among SWE reliable change scores, as well as PDE reliable change scores, and mean FA and MD values for white matter tracts implicated in reading processes were evaluated. Given the small sample size of each group (20–24), we used Bootstrap resampling (1,000 resamples) to generate confidence intervals (BCa, 95%) and interpreted only correlations whose CIs did not include zero. This approach avoids the inflated Type II error risk associated with strict multiple comparison correction and emphasizes effect size estimation (Chan & Chan, 2004).

Further analyses were run for any significantly correlated relations. Individual hierarchical multiple regression models were used to test whether white matter tracts that were significantly correlated with reliable change contributed unique variance to change in reading efficiency following stimulation. Model 1 included WJ Basic Reading SS, FSIQ, age, percentage of total outliers for eddy corrected diffusion data and global white matter volume as predictors of RCI. Proceeding models included mean FA or MD values for white matter tracts that were significantly correlated with reliable change scores.

## RESULTS

### Preliminary Analysis

Descriptive statistics and demographic data for participants included in final analyses are reported in Table 3. As anticipated, there were no significant differences between groups on age, FSIQ, or baseline reading ability. FDR corrected correlations were assessed for individual baseline reading measures across diffusivity metrics for tracts of interest (i.e., single behavioral measure correlated with all metrics for all tracts). Significant correlations were found with regions of the body of the CC, including the rostrum MD (*r* = 0.36, *p* = 0.019) and body genu MD (*r* = 0.31, *p* = 0.036) and WJIV Basic Reading standard score. Significant correlations were also found with FA values of the left (*r* = 0.35, *p* = 0.020) and right (*r* = 0.42, *p* = 0.007) AF, with the left (*r* = 0.34, *p* = 0.022) and right (*r* = 0.39, *p* = 0.013) SLF-II, as well as with the left (*r* = 0.37, *p* = 0.015) and right (*r* = 0.42, *p* = 0.007) SLF-III. Additionally, significant correlations were found with the left UF FA (*r* = 0.30, *p* = 0.045), right UF FA (*r* = 0.37, *p* = 0.015) and right UF MD (*r* = 0.33, *p* = 0.027).

**Table 3.** Demographics of study participants by stimulation site

Stimulation site	Left temporoparietal		Right temporoparietal		Vertex	
	*N*	Mean (*SD*)	*N*	Mean (*SD*)	*N*	Mean (*SD*)
Female (male)	14 (6)		15 (9)		13 (9)	
Age at baseline MRI		24.20 (6.99)		23.28 (6.15)		21.60 (3.81)
ARHQ score		0.33 (0.13)		0.36 (0.19)		0.31 (0.13)
FSIQ		113.25 (13.69)		113.52 (12.15)		115.52 (12.52)
WJIV Basic Reading SS		99.85 (11.01)		101.69 (12.11)		107.30 (11.46)

*Note*: ARHQ = Adult Reading History Questionnaire; FSIQ = Full-Scale Intelligence Quotient; WJIV Basic Reading SS = Woodcock-Johnson Basic Reading Cluster standard score.

Baseline TOWRE-2 SWE (Form A) was significantly correlated with CC body prefrontal FA (*r* = 0.31, *p* = 0.035), body temporal FA (*r* = 0.37, *p* = 0.023), genu FA (*r* = 0.41, *p* = 0.012), and rostrum FA (*r* = 0.32, *p* = 0.035). Significant correlations were also found with FA values of the left (*r* = 0.34, *p* = 0.035) and right (*r* = 0.34, *p* = 0.035) AF, left (*r* = 0.31, *p* = 0.037) and right (*r* = 0.30, *p* = 0.041) ILF, as well as left (*r* = 0.32, *p* = 0.035) and right (*r* = 0.33, *p* = 0.035) SLF-II and the right SLF-III (*r* = 0.29, *p* = 0.043). Additionally, significant correlations were found with the left UF FA (*r* = 0.30, *p* = 0.037), right UF FA (*r* = 0.41, *p* = 0.012), and right UF MD (*r* = 0.29, *p* = 0.043). There were no significant correlations between Baseline TOWRE-2 PDE (Form A) and the mean FA and MD for tracts of interest.

**Figure 2.**
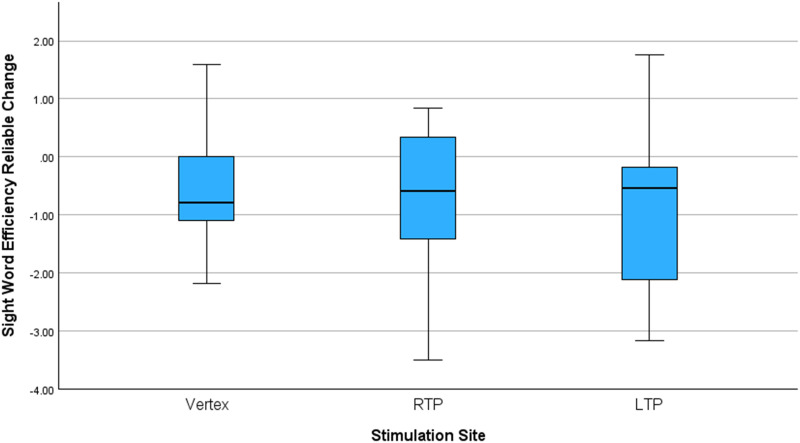
Sight word efficiency reliable change index by stimulation site. LTP = left temporoparietal region; RTP = right temporoparietal region.

Stimulation site differences in reliable change measures are reported in Table 4. Mean RCI in SWE was observably lower, indicating decreased reading performance, when participants received stimulation to the left TPJ (*M* = −0.88, *SD* = 1.21) compared with when participants received stimulation to the right TPJ (*M* = −0.66, *SD* = 1.22) or the vertex (*M* = −0.67, *SD* = 0.86), as shown in Figure 2. However, these mean differences in SWE RCI between stimulation sites did not reach statistical significance (*F*(2, 64) = 0.30, *p* = 0.74). Additionally, no significant mean differences between stimulation sites were observed for PDE RCI (*F*(2, 64) = 0.12, *p* = 0.88).

**Table 4.** Mean reliable change index for TOWRE-2 by stimulation site

Stimulation site	Left temporoparietal	Right temporoparietal	Vertex
*N*	20	24	23
RC *N* SWE/PDE	6/2	4/0	2/2
SWE Mean RCI (*SD*)	−0.88 (1.21)	−0.66 (1.22)	−0.67 (0.86)
PDE Mean RCI (*SD*)	−0.16 (1.14)	−0.12 (0.60)	−0.15 (1.12)

*Note*: PDE = TOWRE-2 Phonemic Decoding Efficiency; RCI = reliable change index; RC *N* = total number of participants in group meeting reliable change criteria (<−1.96 or >1.96); SWE = TOWRE-2 Sight-Word Efficiency.

### Correlational Analyses

Mean FA values of several white matter tracts of interest were significantly correlated with RCI in SWE following stimulation to the left TPJ. Specifically, significant negative correlations, which showed more negative RCI values associated with more positive FA values, were found with regions of the body of the CC, including the body prefrontal (*r* = −0.49, *p* = 0.029), body temporal (*r* = −0.64, *p* = 0.002), and body genu (*r* = −0.52, *p* = 0.019). Significant negative correlations were also found with the left (*r* = −0.61, *p* = 0.004) and right (*r* = −0.50, *p* = 0.025) AF, the left ILF (*r* = −0.48, *p* = 0.031), the right (*r* = −0.46, *p* = 0.043) and left (*r* = −0.47, *p* = 0.037) SLF-II, and the right SLF-III (*r* = −0.49, *p* = 0.030). There were no significant correlations between RCI for SWE and MD values for white matter tracts associated with the reading network. No significant correlations were observed for RCI for PDE and either FA or MD values. No significant correlations were observed for RCI for SWE or PDE following stimulation to right TPJ or vertex.

### Regression Analyses

To test for assumptions of normality, the unstandardized residuals of RCI for SWE were plotted against the predictors in the regression. The plots were visually inspected, and no violations of linearity or homoscedasticity were identified. Across all models, skew and kurtosis of the residuals were acceptable. There was, however, some evidence of leverage. A few data points exhibiting moderate leverage, with leverage values above 0.2, but lower than 0.4.

For regression analyses, 10 separate models were computed (see Table 5). The overall model for Model 1, which included five covariates as predictors of change (age, FSIQ, WJ Basic Reading SS, average global white matter volume, and eddy corrected percentage of total outliers QC variable) was not significant (*F*(5, 14) = 1.87, *p* = 0.164). Average global white matter volume was the only predicter that contributed to a significant percentage of variance in the model (*t* = −2.22, *p* = 0.043). Therefore, subsequent models included average global white matter volume and each of the mean FA values for white matter tracts that were significantly correlated with reliable change in SWE.

**Table 5.** Regression models for tracts of interests predicting SWE RCI

	*β*	*SE*	*t*	*p*	Unique *R*^2^	*F*	*R* ^2^
Model 1: Covariates							
Overall model						1.87	0.40
Age	−0.01	0.04	0.15	0.885	0.00		
FSIQ	0.002	0.02	0.86	0.404	0.03		
WJ Basic Reading SS	−0.04	0.03	−1.40	0.183	0.08		
Global WM volume	−1.05 × 10^−5^	0.00	−2.22	0.043	0.21		
Eddy corrected % QC	−2.4	2.2	−1.07	0.303	0.05		

Model 2: CC (body prefrontal)							
Overall model						6.77	0.44
Global WM volume	−9.66 × 10^−6^	0.00	−2.50	0.023	0.20		
CC (body prefrontal)	−15.32	6.47	−2.37	0.030	0.18		

Model 3: CC (body temporal)							
Overall model						10.17	0.55
Global WM volume	−7.96 × 10^−6^	0.00	−2.23	0.040	0.13		
CC (body temporal)	−31.57	9.67	−0.55	0.005	0.29		

Model 4: CC (genu)							
Overall model						9.37	0.52
Global WM volume	−1.07 × 10^−5^	0.00	−3.01	0.008	0.25		
CC (body genu)	−17.99	5.85	−3.08	0.007	0.27		

Model 5: Left AF							
Overall model						6.82	0.45
Global WM volume	−6.27 × 10^−6^	0.00	−1.47	0.160	0.07		
Left AF	−20.95	8.78	−2.39	0.029	0.19		

Model 6: Right AF							
Overall model						6.36	0.43
Global WM volume	−9.15 × 10^−6^	0.00	−2.31	0.033	0.18		
Right AF	−19.51	8.71	−2.24	0.039	0.17		

Model 7: Left ILF							
Overall model						5.93	0.41
Global WM volume	−9.11 × 10^−6^	0.00	−2.26	0.037	0.18		
Left ILF	−18.42	8.81	−2.09	0.052	0.15		

Model 8: Left SLF II							
Overall model						4.97	0.37
Global WM volume	−8.56 × 10^−6^	0.00	−2.00	0.062	0.15		
Left SLF II	−14.36	8.36	−1.72	0.104	0.11		

Model 9: Right SLF II							
Overall model						6.12	0.42
Global WM volume	−9.78 × 10^−6^	0.00	−2.48	0.024	0.21		
Right SLF II	−17.07	7.91	−2.16	0.046	0.16		

Model 10: Right SLF III							
Overall model						7.78	0.48
Global WM volume	−1.04 × 10^−5^	0.00	−2.80	0.012	0.24		
Right SLF III	−19.63	7.36	−2.67	0.016	0.22		

*Note*: AF = arcuate fasciculus; CC = corpus callosum; FSIQ = Full-Scale Intelligence Quotient; ILF = inferior longitudinal fasciculus; RCI = reliable change index; SLF = superior longitudinal fasciculus; SWE = TOWRE-2 Sight-Word Efficiency; WJ Basic Reading SS = Woodcock-Johnson Basic Reading Cluster standard score; WM = white matter.

Independent models predicting RCI for SWE following stimulation of the left TPJ were conducted. Each model included average global white matter volume and mean FA for one of the following: CC body prefrontal, CC body temporal, CC body genu, left AF, right AF, left ILF, left SLF II, right SLF II, or right SLF III, plus baseline SWE score as predictors. All overall models were significant, as follows: (M2) CC − body prefrontal: 44% (*F*(2, 17) = 6.77, *p* = 0.007); (M3) CC − body temporal: 55% (*F*(2, 17) = 10.17, *p* = 0.001); (M4) CC − body genu: 52% (*F*(2, 17) = 9.37, *p* = 002); (M5) left AF: 45% (*F*(2, 17) = 6.82, *p* = 0.007); (M6) right AF: 43% (*F*(2, 17) = 6.36, *p* = 0.009); (M7) left ILF: 41% (*F*(2, 17) = 5.76, *p* = 0.011); (M8) left SLF II: 37% (*F*(2, 17) = 4.97, *p* = 0.020) (M9) right SLF II: 42% (*F*(2, 17) = 6.12, *p* = 0.010); and (M10) right SLF III: 48% (*F*(2, 17) = 7.78, *p* = 0.004).

In the left ILF model (Model 7), only average global white matter volume contributed unique variance to SWE RCI scores, (*β* = −9.15 × 10^−6^, *p* = 0.037, unique *R*^2^ = 0.18). In contrast, in the left AF model (Model 5), only mean FA for the left AF contributed significant variance to SWE RCI scores.

In the left SLF II model (Model 8) neither the mean FA for the left SLF II nor the average global white matter volume accounted for unique variance. Mean FA for the following tracts of interest accounted for the significant variance in SWE RCI: (M2) CC − body prefrontal: (*β* = −15.32, *p* = 0.030, unique *R*^2^ = 0.18); (M3) CC − body temporal: (*β* = −31.57, *p* = 0.005, unique *R*^2^ = 0.29); (M4) CC − body genu: (*β* = −17.99, *p* = 0.007, unique *R*^2^ = 0.27); (M5) left AF: (*β* = −20.95, *p* = 0.029, unique *R*^2^ = 0.19); (M6) right AF: (*β* = −19.51, *p* = 0.039, unique *R*^2^ = 0.17); (M9) right SLF II: (*β* = −17.07, *p* = 0.046, unique *R*^2^ = 0.16); and (M10) right SLF III: (*β* = −19.63, *p* = 0.016, unique *R*^2^ = 0.22).

## DISCUSSION

The current study investigated the relation between pre-TMS white matter microstructure and change in timed reading efficiency following stimulation of a key node of the dorsal pathway of the reading network (left TPJ), as well as its right hemisphere homolog, and a control site (vertex). We focused on specific white matter tracts associated with the reading network as predictors of reliable change in sight word and pseudoword reading efficiency. The current study highlights the role of structural connectivity within the reading network and its relation to reading performance. Measures of white matter diffusivity (mean FA) for several tracts of interest accounted for a significant amount of variance in change in sight word reading efficiency following stimulation of a primary node of the dorsal circuit (left TPJ) over and above other behavioral predictors. FA values for regions of the CC (body prefrontal, body temporal, body genu) as well as bilateral AF, and right SLF all accounted for significant proportions of the variance in SWE RCI.

The temporal segment of the body of the CC had the strongest predictive power, with the model explaining 44% of the variance in SWE RCI. This suggests that FA in the CC body temporal is associated with more substantial change in sight word reading following TMS. Although reading and language are primarily left lateralized, this finding aligns with research highlighting the role of the CC in integrating and transferring information between hemispheres, which may be critical for reading processes (De León Reyes et al., 2020). Interhemispheric transfer has been shown to facilitate the development of phonological awareness, a critical skill for decoding written words. Research also indicates that effective interhemispheric communication is necessary for efficient reading (Van der Haegen et al., 2013; Vandermosten et al., 2013). Deficits in interhemispheric transfer may contribute to the difficulties in integrating phonological and orthographic information that are frequently associated with reading impairments characteristic of developmental dyslexia (Katzir et al., 2006). Neuroimaging studies indicate that as reading skills develop, there is increased activation in areas that support interhemispheric communication, such as the corpus callosum (Pugh et al., 2013). This structural adaptation is associated with enhanced reading abilities, which would be supported by our current findings.

Interestingly, the prefrontal and genu body portions of the CC body also showed significant relations with SWE RCI, indicating that the connectivity in these areas may also contribute to the variability in changes in reading following stimulation. Although executive functioning has historically been associated with frontal cortical regions (Marek & Dosenbach, 2019), recent evidence suggests that regions of the CC play an important role in supporting these cognitive processes, with CC microstructure independently contributing to executive functioning (Bettcher et al., 2016). This may suggest that connectivity in regions associated with executive function and higher cognitive processes may be involved in facilitating change in reading following TMS. Given that executive functions such as working memory and inhibitory control are necessary for efficient reading, it follows that anterior brain structures associated with these skills are also shown to support reading (Alloway & Copello, 2013; Swanson & Sachse-Lee, 2001). Results of the current study would suggest that communication between these critical brain regions that is supported by connections via the CC may support basic word recognition and reading efficiency.

Bilateral AF also showed significant predictive power, with the model explaining up to 45% of the variance in change in reading following TMS. These findings are consistent with the role of the AF in language processing and its involvement in the neural networks supporting reading and language abilities (Catani et al., 2005). Specifically, the AF is a crucial white matter tract that connects the dorsal pathway with the anterior pathway and plays a significant role in language processing, including single word reading. The AF supports the retrieval of lexical information, allowing for the quick access of stored word meanings and pronunciations, which is necessary for fluent reading of written words (Duffau, 2008). Efficient communication via the AF is also believed to contribute to reading efficiency by helping to link orthographic and phonological processing during single-word reading tasks such as the one used in this study (Gullick & Booth, 2015; Yeatman et al., 2011). Neural modulation resulting from TMS to a node of the dorsal pathway may have downstream network effects due to its propagation down the AF. As such, diffusivity properties of this tract may play a role in the change in reading behavior that is observed following stimulation.

Parietal portions of the right SLF (SLF-II) was also a significant predictor of reliable change in SWE following stimulation to the left TPJ. These results further support the involvement of this tract in reading processes (Zhang et al., 2014). Like the AF, the connection between the dorsal and anterior pathways, via the SLF, is important for facilitating the retrieval of lexical information to allow for efficient single word reading (Bernal & Altman, 2010). Diffusivity of the SLF has also been linked to the acquisition of reading with increased FA positively correlating with better reading abilities (Cheema & Cummine, 2018). Parietal segments of the SLF connect occipital and parietal cortical regions with Broca’s area to help integrate visual and language processing that is essential for fluent reading. In combination with the AF, portions of the SLF serve as critical connections within the dorsal pathway for supporting efficient reading skills. Results of the current study highlight this in that the observed relation between white matter diffusivity and change in reading following stimulation suggest that these connections underlie necessary communication within the network to facilitate reading.

While portions of the CC and tracts associated with the dorsal circuit of the reading network (i.e., AF and SLF) were the strongest predictors of change, the model including left ILF was also significant, with the FA of the ILF trending toward significance. This could be due, in part, to its primary connections from the occipital cortex to the inferior temporal gyrus and visual word form area (Epelbaum et al., 2008). Additionally, the ILF has been shown to have parietal projections that terminate in the inferior parietal lobule (Bajada et al., 2015; Schmahmann & Pandya, 2006). Although the role of the ILF in language processing is poorly understood, research supports its role in the ventral reading circuit (Arrington et al., 2017; Horowitz-Kraus et al., 2014), with damage producing impairments in word recognition (Epelbaum et al., 2008; Ng et al., 2021). Direct electrical stimulation of the left ILF has been shown to impair visual semantic but not phonological processing, supporting the role of this tract in visual word recognition (Papatzalas et al., 2022). Our findings suggesting a relation between the left ILF and change in sight word but not pseudoword reading would also support the role of this tract in word recognition. Behavioral change following stimulation to the left TPJ may be partly related to downstream signal propagation via the ILF through its parietal fiber projections.

In addition to underscoring the role of white matter tracts in supporting reading, results of the current study also highlight the importance of considering individual differences in white matter diffusivity in the utilization of neuromodulation as a possible treatment for reading impairments. Functional and anatomical neuroimaging studies have demonstrated compromised connectivity among brain regions in reading impairments such as developmental dyslexia (Niogi & McCandliss, 2006; Odegard et al., 2009; Paulesu et al., 1996). Additionally, readers with dyslexia have shown atypical activation in the left TPJ region along with an increased activation of the corresponding right hemisphere which is further correlated with deficits in phonological processing (Breier et al., 2003). Theories regarding the neurobiological basis of dyslexia range from problems with hypoarousal (Pugh et al., 2008) to the effects of hyperarousal or noise (Hancock et al., 2017) in the reading network. TMS may be a useful tool not only for understanding the basis for such neural mechanisms supporting reading, but also as an intervention component for treatment resistant reading impairment (van den Noort et al., 2015).

Although no significant mean differences in behavioral change indices were observed following stimulation of either the left or right TPJ, relative to the control site, the largest change was observed following stimulation to the left TPJ. This may be somewhat related to reliability and measurement qualities of the timed reading measure utilized (Tanna, 2009). Additionally, the variable responses to stimulation observed in the current data (i.e., both facilitation and suppressive effects) could account for a washout effect in site related differences. Although cTBS has traditionally been thought of as an inhibitory stimulation protocol (Huang et al., 2005), recent evidence suggests that these effects are not consistent in the reading network (Harrington et al., 2023). Theta burst stimulation, cTBS in particular, is also highly sensitive to interindividual variability (Jannati et al., 2017; Lowe & Hall, 2018). This may account for the variable response observed in the current study.

Despite this, previous literature has shown that TMS to a targeted node of the reading network can effectively modulate component reading skills (see Arrington et al., 2022, for review). High frequency repetitive TMS (rTMS) has also be shown to improve reading in individuals with dyslexia (Costanzo et al., 2013; Turker & Hartwigsen, 2022). Results of the current study suggest that behavioral reading change following stimulation may be related to structural connectivity within the network. Our initial model, which included global white matter volume, suggested a trend of a relationship between whole brain diffusivity characteristics and reading; however, this metric was only significant in one of the proceeding models in which the tract specific FA was not a significant predictor (Model 7, left ILF). We were unable to directly assess the contribution of whole brain FA due to limitations of our tractography methodology but believe that the anatomically informed approach provided by TRACULA is ideal when assessing the contribution of individual tracts that make up the complex reading network (Andica et al., 2023). Individual network components, rather than whole brain characteristics, have been shown to relate to distinct components of reading fluency such as single word reading efficiency (Zhang et al., 2014). This is highlighted by our findings supporting a relationship between white matter diffusivity and the levels of change in reading efficiency scores but only following stimulation of a direct node of the reading network (i.e., left TPJ). Although limitations in diffusion data acquisition (i.e., single shell with high *b* values) may not prove ideal for the tensor fitting methodology used, we believe that results of the current study support this. Similarly, previous literature suggests that properties of white matter diffusivity have been shown to influence both functional connectivity (Boorman et al., 2007; Kearney-Ramos et al., 2018) and behavioral outcomes (Klöppel et al., 2008; Quentin et al., 2013) following stimulation. This would suggest that downstream effects of TMS may be heavily dependent on structural connectivity within the network, particularly in complex networks such as those supporting reading.

Differences in white matter diffusivity between impaired and typically skilled readers also highlight the importance of understanding structural connectivity as it relates to change in reading behavior. Reduced FA values within the SLF have been reported in adults with poor word reading ability (Carter et al., 2009; Vandermosten, Boets, Poelmans, et al., 2012). Voxel by voxel comparison of FA values in impaired versus typical readers has shown that there are significant bilateral differences in temporoparietal white matter regions with a significant correlation between reading skills and FA between the groups, suggesting a reading deficit due to reduced white matter (Klingberg et al., 2000). Differences in micro- and macrostructural properties of posterior regions of the CC have also been reported in children and adults with reading impairments (Hasan et al., 2012; Rumsey et al., 1996; von Plessen et al., 2002). Given the relationship between white matter diffusivity and change in reading efficiency observed in the current study, understanding baseline structural connectivity may be important for predicting the effects of neuromodulation on impaired reader populations. This is of particular importance given that evidence suggests that behavioral reading instruction alone has been shown to change morphological properties of white matter tracts associated with reading in both typical and impaired readers (Castro-Caldas et al., 1999; Gebauer et al., 2012; Keller & Just, 2009; Takeuchi et al., 2016). For treatment-resistant reading impairments, eliciting this neuroplastic change with rTMS prior to reading instruction may help to facilitate the desired behavioral change.

Overall, these findings contribute to the understanding of how structural connectivity in white matter tracts influence changes in reading efficiency following rTMS. The results underscore the importance of specific white matter pathways in modulating the effects of TMS on reading skills and suggest that targeted interventions might be optimized by considering individual differences in white matter connectivity.

## ACKNOWLEDGMENTS

The authors would like to acknowledge Dr. Ayan Mitra, Dr. Zhichao Xia, Yishai Ponce Perez, and Flora Heydari for help with data collection. Authors would also like to thank Dr. Roeland Hancock and Dr. Vishwadeep Ahluwalia for imaging sequence development.

## FUNDING INFORMATION

Fumiko Hoeft, National Institute of Child Health and Human Development (https://dx.doi.org/10.13039/100000071), Award ID: R01HD096261.

## AUTHOR CONTRIBUTIONS

**C. Nikki Arrington**: Investigation: Equal; Methodology: Lead; Project administration: Equal; Writing – original draft: Lead. **Ewelina Bledniak**: Investigation: Equal; Writing – original draft: Supporting. **Brianna Kinnie**: Data curation: Lead; Project administration: Supporting; Writing – original draft: Supporting. **Evelyn Farkas**: Investigation: Supporting; Writing – original draft: Supporting. **Robin Morris**: Conceptualization: Supporting; Funding acquisition: Supporting; Resources: Lead; Writing – review & editing: Equal. **Fumiko Hoeft**: Funding acquisition: Lead; Supervision: Supporting; Writing – review & editing: Equal.

## DATA AND CODE AVAILABILITY STATEMENTS

All data and analysis code supporting the findings of this study are openly available on the Open Science Framework (OSF): https://doi.org/10.17605/OSF.IO/2TDJ4. The preprocessing scripts, statistical analyses, and visualization code are archived under a CC-BY 4.0 license. Neuroimaging data is available from the corresponding author upon reasonable request via the Collaborative Informatics and Neuroimaging Suite Data Exchange.

## TECHNICAL TERMS

Transcranial magnetic stimulation (TMS): A noninvasive brain stimulation technique that uses magnetic fields to stimulate neurons in the targeted brain region.
Continuous theta burst stimulation (cTBS): A type of repetitive transcranial magnetic stimulation (50 Hz) that uses patterned bursts of stimulation to induce changes in cortical excitability.
Diffusion weighted imaging (DWI): A magnetic resonance imaging technique that uses the random motion of water molecules to generate contrasts to quantify white matter pathways.
